# Kisspeptin a potential therapeutic target in treatment of both metabolic and reproductive dysfunction

**DOI:** 10.1111/1753-0407.13541

**Published:** 2024-04-10

**Authors:** Joanna Helena Sliwowska, Nicola Elizabeth Woods, Abdullah Rzgallah Alzahrani, Elpiniki Paspali, Rothwelle Joseph Tate, Valerie Anne Ferro

**Affiliations:** ^1^ Department of Veterinary Medicine and Animal Sciences, Laboratory of Neurobiology Poznan University of Life Sciences Poznan Poland; ^2^ Strathclyde Institute of Pharmacy and Biomedical Sciences University of Strathclyde Glasgow UK; ^3^ Department of Pharmacology and Toxicology, Faculty of Medicine Umm Al‐Qura University Makkah Saudi Arabia; ^4^ Department of Chemical Engineering University of Strathclyde Glasgow UK

**Keywords:** diabetes mellitus, KISS1/Kiss1, KISS1R/Kiss1r, kisspeptin, obesity, reproduction

## Abstract

Kisspeptins (KPs) are proteins that were first recognized to have antimetastatic action. Later, the critical role of this peptide in the regulation of reproduction was proved. In recent years, evidence has been accumulated supporting a role for KPs in regulating metabolic processes in a sexual dimorphic manner. It has been proposed that KPs regulate metabolism both indirectly via gonadal hormones and/or directly via the kisspeptin receptor in the brain, brown adipose tissue, and pancreas. The aim of the review is to provide both experimental and clinical evidence indicating that KPs are peptides linking metabolism and reproduction. We propose that KPs could be used as a potential target to treat both metabolic and reproductive abnormalities. Thus, we focus on the consequences of disruptions in KPs and their receptors in metabolic conditions such as diabetes, undernutrition, obesity, and reproductive disorders (hypogonadotropic hypogonadism and polycystic ovary syndrome). Data from both animal models and human subjects indicate that alterations in KPs in the case of metabolic imbalance lead also to disruptions in reproductive functions. Changes both in the hypothalamic and peripheral KP systems in animal models of the aforementioned disorders are discussed. Finally, an overview of current clinical studies involving KP in fertility and metabolism show fewer studies on metabolism (15%) and only one to date on both. Presented data indicate a dynamic and emerging field of KP studies as possible therapeutic targets in treatments of both reproductive and metabolic dysfunctions.

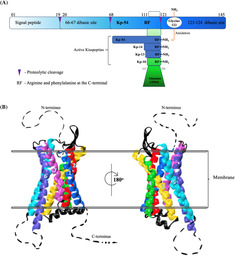

## Graphical abstract

- Kisspeptins (KPs) were first discovered to have antimetastatic action. Historically, a role in reproduction was demonstrated. Today, there is growing experimental and clinical evidence that KPs and their receptors regulate metabolism.
- Disruption of KP and receptor interaction provides an interesting approach to treating metabolic conditions, as well as reproductive disorders.
- Clinical trials have focused mainly on treating reproductive dysfunctions; however, greater understanding of the biology and mechanisms of action of KPs is being used in the therapeutic armory against insulin‐related dysfunctions.
- Linkage between reproduction and metabolism via the KP system also allows for therapeutic targeting.

## 1 INTRODUCTION

### 1.1 Emerging functions of kisspeptins (KPs)

The role of kisspeptins (KPs) was first investigated in the field of oncology as an antimetastatic factor, known as metastin, later in reproductive biology, and more recently in the control of metabolic functions.^1^, ^2^, ^3^ Their effect has an impact throughout the body, including the brain, reproductive organs, pancreas, cardiovascular system, and kidneys.^4^

The unraveling of the complexities of this family of peptides in the regulation of reproduction began with the concept of the existence of hypothalamic releasing hormones and subsequent gonadotropin‐releasing hormone (GnRH).^5^ However, it was recognized that GnRH was not the sole regulator of reproductive function, particularly as satisfactory explanations could not be found for the pulsatile release of GnRH and the onset of puberty. Hence, some other factor(s) had to be involved. Growing evidence from genetic loss‐of‐function variant studies in the early 2000s led to the understanding of the involvement of KPs in GnRH release^6^ and the regulation of puberty.^7^, ^8^, ^9^ This was surprising because knowledge of KP and the *KISS1* gene was already established in an antimetastasis role.^10^

### 1.2 KP and its receptor

**FIGURE 1:**
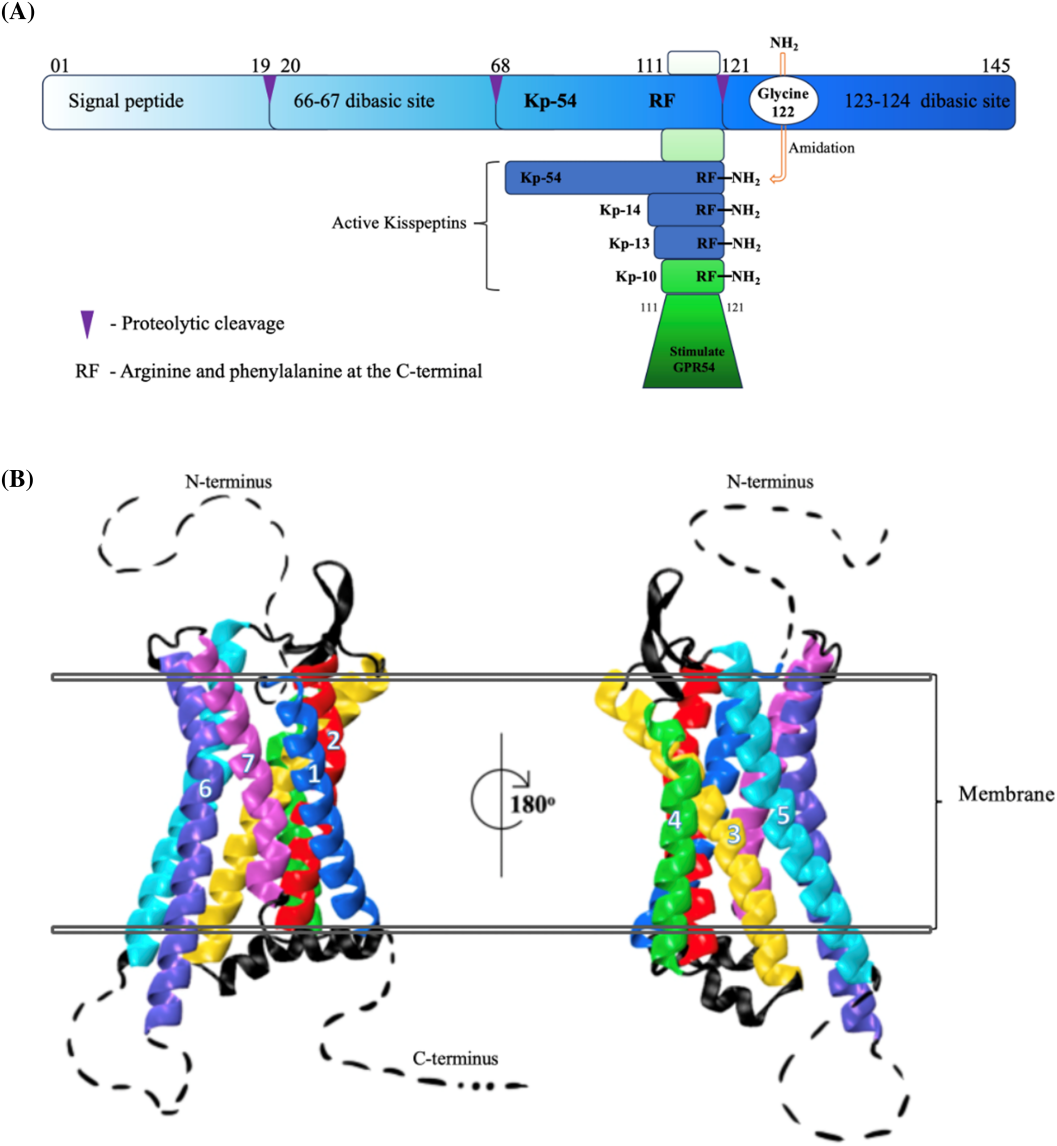
Kisspeptin and the human GPR54 structure. (A) Schematic diagram showing pre‐pro‐kisspeptin and the proteolytic cleavage pathway from which active kisspeptins Kp‐10, Kp‐13, Kp‐14, and Kp‐54 occur. (B) The 3‐dimensional Cryo‐EM structure of CPR54 (SWISS‐MODEL, SMLT ID: 7xjk.1).^12^ The membrane bilayer is indicated by the black double lines and the transmembrane helices of CPR54 and colored and numbered (Helix1: blue, Helix2: red, Helix3: yellow, Helix4: green, Helix5: cyan, Helix6: purple, and Helix7: pink), whereas the extra‐ and intracellular loops are depicted with black color. The dashed lines indicate the missing segments of the GPR54 reported crystal structure. Image was made using the VMD software.^13^ GPR54, G‐protein‐coupled receptor; KP, kisspeptin.

The gene for KP was named *KISS1* as it was first cloned in Hershey, Pennsylvania, a city known for making chocolate kisses.^11^ The 54 amino acid peptide, Kisspeptin‐54 (Kp‐54), is the major known fraction. Figure 1 shows how different fractions of KPs are derived from the first transcription of *KISS1*, which consists of 145 amino acids (pre‐pro‐kisspeptin) that are then proteolytically cleaved to produce Kp‐54 and the less common Kp‐14, Kp‐13, and Kp‐10 peptides.^14^

*KISS1* mRNA is widely expressed in numerous tissues including the placenta,^15^ testis,^16^ ovaries,^17^ liver,^18^ pancreas,^19^ small intestine,^7^ and crucially in the central nervous system (CNS), in particular the hypothalamus, where KISS1 is a key regulator in fertility.^20^, ^21^ It is important to note that in the hypothalmus, expression of kisspeptin and its receptor varies considerably depending on the species and gender and throughout the life stage of an organism.^22^ Aligned with its role in the development and regulation of cancers, KISS1 and its receptor are also found in a number of human cancers including breast and prostate, where they can serve as both prognostic biomarkers and therapeutic targets.^23^ The receptor is classed as a G‐protein coupled receptor (GPCR) with weak homology to galanin receptors.^24^ The human KP receptor (KISS1R) is also known as AXOR12, HH8, and hOT7T175, with the most common name being GPR54.^25^ The way in which the KPs interact with the receptor has been reviewed by Trevisan et al.^14^

### 1.3 Role of KP in reproduction

Fertility and reproductive function are regulated via the hypothalamic–pituitary‐gonadal axis (HPG). This is a complex neurohormonal system of control, driven by GnRH in the hypothalamus, luteinizing hormone (LH), and follicle‐stimulating hormone (FSH) in the pituitary and then steroids in the gonads.

Although known in the 1970s that the initiation of mammalian sexual maturation requires the activation of the hypothalamic GnRH neurons,^26^ the mechanism responsible for it remained unknown. The key role of KP in the stimulation of GnRH neurons and, consequently, in the initiation of sexual maturation has been demonstrated by scientists led by the De Roux group,^27^ on what was then referred to as family‐related idiopathic hypogonadotrophic hypogonadism (IHH). In the siblings of an examined child, four out of five brothers developed symptoms of IHH (characterized as undeveloped testes, no pubic hair, and a 15‐year‐old skeletal system), and one of two sisters showed symptoms of partial hypogonadism (her breasts developed only partially and up to the age of 16 had only had one menstrual period). Each of his siblings had low blood levels of gonadotropins and gonadal hormones, and further tests showed that the family had a *KISS1R* gene variant. Based on these studies it was concluded that the KISS1/KISS1R system is responsible for initiating sexual maturation. Further, the research of Seminara et al,^8^ who diagnosed the syndrome of IHH in six members (four men and two women) of a family, showed it was due to a *KISS1* gene variant. The importance of the KISS/KISS1R system in the proper functioning of the HPG axis was also confirmed by later work of this group on mice with a *Kiss1r* gene variant. Thus, KP is essential in the initiation of puberty. KISS1R activates phospholipase C (PLC) when it is coupled to the G protein subunit, G_q/11_α.^14^ PLC activation causes phosphatidylinositol bisphosphate to be hydrolyzed and form diacylglycerol (DAG).^28^ DAG activates protein kinase C, which phosphorylates mitogen‐activated protein kinases that then serve as activators for β‐arrestin and inositol‐1,4,5‐triphosphate.^29^ As a result of these cascades, calcium is released intracellularly to depolarize the neuron.^30^ Calcium levels may increase intracellularly as a result of PLC‐independent mechanisms, leading to opening potassium channels and nonselective cation channels.^31^ Synapses are formed between KP and GnRH expressing neurons and KP neurons are depolarized, as are GnRH neurons. This depolarization causes release of LH and FSH.^32^, ^33^ Prolonged exposure to KP desensitizes KISS1R.^32^ KP administration stimulates gonadotropin secretion in a wide range of species including rodents, goats, sheep, pigs, cows, horses, and monkeys^34^ as well as humans.^35^, ^36^, ^37^

In the peripheral reproductive system there is evidence that KP and KISS1R play a role in local control of reproductive function within the ovary, testis, uterus, and placenta.^38^ Furthermore, KP is involved in all stages of reproduction including onset of puberty, menstrual cycle, pregnancy and in the menopause.^14^, ^39^ The response of KP in both genders is different and the roles of KP and GnRH are reviewed comprehensively by Marquez et al.^40^

### 1.4 KP does not act alone

Within the hypothalamic arcuate nucleus (ARC), KP acts together with two cotransmitters: neurokinin B (NKB) and dynorphin (Dyn). The neurons responsible in the ARC are called KNDy (from the first letter of peptides: K – kisspeptin, N – neurokinin B, D – dynorphin).^41^ In rodents, NKB is a tachykinin peptide that binds to the NK3R.^42^ TAC3 is the name for NKB in humans, which acts on TAC3R.^43^, ^44^ Dyn belongs to a class of endogenous opioid peptides that act on opioid kappa receptors.^45^ In the hypothalamus, KP release is stimulated by NKB and inhibited by Dyn.^46^ However, there are species differences in the colocalization of the aforementioned peptides, and a low degree of overlap between KP, NKB, and Dyn immunoreactivities found in the infundibular recess (equivalent to the ARC) of young men challenges KNDy neuron concepts in humans.^47^ For further information, refer to the comprehensive review of the KNDy hypothesis by Lehman et al.^48^ In mammals, besides the population of KP neurons in the ARC there is another population of KP‐synthesizing neurons in the anterior preoptic area. In rodents, KP neurons are located in two main populations of the rostral periventricular area of the third ventricle (RP3V) and in the ARC.^48^ In humans, both KP cell populations are also present, with the majority of neurons localized in the infundibular nucleus.^49^, ^50^ Preovulatory surge of GnRH and LH are controlled by the first group of cells, and pulsatile LH release is governed by the second one that projects onto GnRH. Estradiol (E_2_) works in the opposite manner on both populations of KP neurons. Whereas in the RP3V, KP synthesis^51^ is enhanced by E_2_, in the ARC, estradiol inhibits the synthesis of KP. Our understanding of the KNDy neuronal network is critical to identifying new roles for KP, not only in reproduction.

### 1.5 KP as a neuropeptide linking metabolism and reproduction and possible tool to treat both metabolic and reproduction dysfunction

Although KP control of reproduction is very well established, its involvement in metabolism regulations is still emerging. However, there are also interconnections between these two processes.

First, reproduction, which is necessary for the perpetuation of the species, is very costly in terms of energy consumption. Maturation and function of the HPG axis are tightly connected with the energy status of an individual.^52^ Historically, in paleolithic ages, symbols of fertility were presented as overweight women. Much later, in the 1960s and 1970s, the critical fat mass hypothesis was formulated that stated a need for a certain threshold of body fat mass to be reached in order for the attainment of menarche and for maintaining reproductive function later in life.^53^

However, today there is a worldwide epidemic of obesity, and positive energy balance (especially excessive consumption of foods, high in sugars and fats), reduced physical activity, sedentary lifestyle, and limited physical activity are factors contributing to the development of this metabolic condition.^54^ Moreover, this type of lifestyle promotes development of type 2 diabetes (T2D), which accounts for 90% of diabetes cases. In these patients besides metabolic problems, there are multiple secondary physiological alterations including disruptions of the menstrual cycle, premature childbirth, miscarriages, decrease in testosterone concentrations in men, and fertility impairment in both sexes.^55^, ^56^ In men, obesity is also associated with impaired gonadal function and hypogonadism.^57^ It has been hypothesized that testosterone (T) deficiency reported in obese and T2D patients is connected with decreased endogenous KP secretion and alterations in metabolic and endocrine factors.^58^

In women with polycystic ovary syndrome (PCOS), obesity is common. It is pertinent to note that in Eastern Europe and America, about 50% of women are overweight.^56^, ^59^, ^60^, ^61^, ^62^ KP levels are reportedly higher in women with PCOS, and an overactive KISS system enhances HPG‐axis activity. This in turn results in irregular menstrual cycles and excessive androgen release in these women (reviewed in^63^). Moreover, fertility can be affected by factors such as body weight (BW) and composition, physical activity, and nutrient intake.^64^ Impairment of the HPG axis, oocyte quality, and uterine receptivity have been reported in overweight and obese women.^65^, ^66^ On the other hand, obese men show a total sperm count lower than normal weight controls.^67^ Evidence suggests that physical activity and a proper diet could significantly improve reproductive outcomes.^64^ Additionally, clinical data identify that a KP agonist, MVT‐602, could be a potential drug for treatment of female reproductive disorders.^68^ KP‐10 administration stimulates serum T and LH secretion in men with T2D and mild hypogonadism.^69^ Thus, the data support links between metabolism and reproduction and the role of KP in treatment of these conditions.

The World Health Organization provides the following definitions of being overweight and obese as abnormal or excessive fat accumulation that is a risk to health. Moreover, a body mass index (BMI) >25 kg/m^2^ is categorized as overweight, and >30 as obese.^70^ However, scientific findings indicate that the interplay between reproduction and metabolism occurs within the CNS, in particular in the ARC. Here, several populations of neurons control food intake and metabolism such as neuropeptide Y (NPY), proopiomelanocrtin (POMC), cocaine and amphetamine‐regulated transcript, and a population express agouti‐related peptide.^71^, ^72^ Besides these peptides, there is strong evidence that KP neurons in the ARC play a role in conveying information on metabolism to GnRH neurons being a part of the HPG axis.^52^, ^73^ Murine dual immunofluorescence studies have revealed close appositions between KP fibers and GnRH cell bodies, which increase in number throughout pubertal development.^74^ Recent research has focused on afferent inputs to KP neurons especially in the area of energy balance, where under‐ or severe overnutrition suppresses fertility. It is suggested that KPs induce their metabolic effects indirectly via gonadal hormones and/or directly via the KP receptors in the brain, brown adipose tissue (BAT), and pancreas.^3^ Other evidence for the role of the KP system in metabolic functions comes from *Kiss1r*‐knockout (KO) mice, which exhibit increased adiposity and reduced energy expenditure.

Finally, recent studies indicate a role of KP signaling on sexual behavior^75^ (eg, sexual motivation, copulatory behavior, bonding^76^). The role of hypothalamic KP in metabolism has also been confirmed in numerous animal studies in conditions such as undernutrition, obesity and diabetes, which are discussed subsequently. Additionally, the peripheral role of KP in the control of metabolism, primarily obesity and diabetes, is described next.^77^

### 1.6 What we can learn from animal models of obesity and diabetes about the role of KP in control of metabolism and reproduction and how we can translate this knowledge into the clinic

Many obesity experimental models exist, for example, genetically induced obesity, obesity caused by a high‐fat (HFD), high‐carbohydrate diets, or a combination of both.^78^ Moreover, various animal fetal models of obesity and diabetes exist (reviewed in^79^). The latter are based on the concept of fetal/early programming, according to which early environmental factors can permanently organize or imprint physiological and behavioral systems. Thus, according to these concepts environmental factor(s) such as maternal diet (eg, undernutrition or overnutrition) affect the structure and functions of tissues and organs. This in turn is in agreement with the developmental origins of health and disease approach, leading to lifelong effects, including development of metabolic and reproductive problems in offspring.^80^

Similar to humans, in animal models of obesity, various dysfunctions are observed including raised body fat and triglyceride levels, glucose intolerance, and insulin resistance, which may lead (especially in extended exposure to HFD) to the development of T2D. A commonly used method to induce T2D in animals employs combination of feeding with HFD followed by injections of low doses of streptozotocin (a toxin that destroys the pancreas).^81^ Such animal models mimic the human condition, where T2D is often associated with obesity and a HFD.

Importantly, animal models mimic human findings with regard to reproductive problems reported in the case of obesity; for example, in obese women as well as monkeys and rodents fed with HFD, advanced puberty is observed.^82^, ^83^, ^84^ In search of possible mechanisms responsible for reproductive alterations in times of metabolic imbalance, a role of altered KP signaling, linking metabolism with reproduction is explored. Next we review data on central and peripheral roles of KP in regulation of metabolism and reproduction in animal models of obesity and diabetes. Numerous studies have revealed that in reproductive diseases (eg, hypogonadotropic hypogonadism and PCOS) and metabolic ones (eg, obesity, diabetes, undernutrition) there are alterations in the expression of KP in the hypothalamus.

### 1.7 Alterations in hypothalamic KP expression

Hypothalamic KISS1 directly stimulates GnRH neurons, which express *KISS1R*, and it was identified that patients with IHH (characterized by impaired secretion of gonadotropins FSH and LH) had inactivating polymorphisms in *KISS1R*. These findings were replicated in mice with *Kiss1 and Kiss1R variants*.^8^

Moreover, in a mouse model of PCOS, characterized by hyperandrogenemia, chronic anovulation, cystic ovarian follicles, and LH and hyperpulsatility, changes in KP were reported. These animals showed marked elevated *Kiss1* expression and increased *Kiss1* neuronal activation in the ARC.^85^ In prenatally androgenized (PNA) models of PCOS in female rats, a significant increase in the number of KP‐immunopositive neurons in the ACR was found compared to control females.^86^ Another PNA rat study reported increased *Kiss1* mRNA expression in the ARC.^87^ Besides alterations in expression of *KISS1* in the reproductive disorders there are also convincing results on changes in this system in animal models of metabolic disorders. Quennell et al^73^ showed that leptin deficiency and diet‐induced obesity in mice were associated with reduced *Kiss1* expression in the hypothalamus. This correlates with the concept that a decreased energy status negatively has an effect on fertility. Thus, a reduction in leptin (a hormone controlling food intake) also has a negative effect on fertility. Furthermore, increased leptin levels increase *Kiss1* gene expression. Fu and van den Pol (2010)^74^ showed that Kiss1 directly interacts with anorexigenic POMC neurons in the ARC through Kiss1r on the neurons. In addition, they demonstrated that Kiss1 inhibits NPY neurons in the ARC, indicating that Kiss1 may modulate appetite and energy homeostasis.

### 1.8 Evidence from genetically modified animals

Furthermore, using *Kiss1r* KO mice Tolson et al^88^ demonstrated that they were characterized by sex‐specific differences in metabolic parameters. Whereas KO female mice showed an obese and diabetic phenotype, with increased BW, leptin levels, adiposity, and impaired glucose intolerance, male KO mice had a normal BW and glucose regulation.^60^ However, the reason for these d phenotypes remains unknown. De Bond et al^89^ subsequently found that despite this development, hypothalamic metabolic gene expression involved in the appetite regulating, including neuropeptide Y (Npy), pro‐opiomelanocortin (Pomc), leptin receptor (Lepr), ghrelin receptor (Ghsr), and melanocortin receptors 3 and 4 (Mc3r, Mc4r) remained unaltered. Thus, these changes may be affected by peripheral rather than central influences. In follow‐up developmental studies, Tolson et al^90^ proved that at 6 weeks old (young adult), KO females already show altered adiposity, leptin levels, metabolism, and energy expenditure, despite reported normal BWs.

Sex differences in several metabolic permanents were also reported in mice lacking KP signaling due to global inactivation of Kiss1r also referred to as Gpr54 in some literature^91^ (eg, displaying profound hypogonadism; Gpr54−/−). These animals were compared to Gpr54 null mice with selective reintroduction of Gpr54 expression only in GnRH cells (Gpr54−/−Tg) and preserved gonadal function. Velasco et al^92^ found that in male mice, global elimination of KP signaling manifested itself as decreased BW, suppression of feeding, and increased adiposity, without changes in glucose tolerance. On the other hand, Gpr54^−/−^ female mice had increased BW gain and adiposity and perturbed glucose tolerance, despite reduced food intake. Gpr54−/−Tg rescued mice were characterized by altered BW gain in males and mild altered glucose tolerance in females. When animals were challenged and exposed to HFD, exaggerated BW gain and adiposity in global Gpr54^−/−^ mice of both sexes were observed, with worse glucose tolerance, especially in females. In the case of the rescued Gpr54^−/−^Tg males, intermediate BW gain and feeding profiles and impaired glucose tolerance were reported. On the other hand, rescued Gpr54^−/−^Tg females were similar to controls in many parameters, except for a modest disruption of glucose tolerance following ovariectomy. Based on this study, a global role of KP signaling in the control of BW and metabolic homeostasis was concluded. Moreover, sexually dimorphic effects of KP in the regulation of BW gain, feeding, and responses to HFD were proved. Finally, in animal models of fasting, reduced *Kiss1* mRNA expression in the hypothalamus in rodents were reported.^93^, ^94^ Short‐term undernutrition in male and female prepubertal rats showed reduced whole hypothalamic *Kiss1* mRNA and increased hypothalamic *Kiss1r* mRNA compared with controls.^93^

### 1.9 Changes in peripheral KP expression

There is also evidence of the role of KP besides the brain in regulation of metabolism. *KISS1* and its receptor are expressed in peripheral tissues involved in the control of metabolism such as the pancreas, liver, and fat.^56^ Next we discuss the role of KP in different metabolic organs. Importantly, as KP levels dramatically increase during pregnancy, we also present data linking alterations in this peptide with gestational diabetes.^95^

#### 1.9.1 KP action in the pancreas

In the pancreas, the 54 amino acid KP isoform, Kp‐54, increases glucose‐induced insulin secretion from human and mice islets, without changing basal secretion.^46^, ^96^ Additionally, studies in rats have also found that an intracerebroventricular injection of the peptide had no effect on insulin levels, which confirms a peripheral site of action of Kp‐54.^97^ Moreover, it was revealed that Kp‐10 and Kp‐13 act directly on β‐cells to potentiate insulin secretion stimulated by glucose in murine, porcine, and human islets.^97^, ^98^ Finally, the expression of *KISS1* and *KISS1R* in the pancreas (mRNA and peptide) is altered by an HFD and T2D.^99^

#### 1.9.2 KP action in the liver

In the liver, KP‐10 administered peripherally had antioxidant and thus protective effects on liver metabolism.^18^, ^46^, ^100^, ^101^ In diabetic mice (HFD‐induced diabetes and genetic leptin receptor‐deficient db/db animals) liver KP levels were increased.^18^ Additionally, obese male rats fed a HFD and those with T2D also had increased liver KP expression.^99^ Importantly, studies in patients with T2D revealed increased liver and plasma KP levels. The role of KP in the regulation of metabolism was also revealed in genetically modified animals. In a selective liver KP knockdown mouse model, depressed glucose‐sensitive insulin secretion and improved glucose tolerance were found. Moreover, mice lacking liver *Kiss1r* when fed a HFD had improved glucose tolerance.^18^

In nonalcoholic fatty liver disease, that correlates with a rise in obesity and T2D, activation of the Kiss1r signaling pathway had therapeutic effects in HFD‐fed C57BL/6J mice. Whereas in these animals, a deletion of hepatic *Kiss1r* exacerbated hepatic steatosis, whereas stimulation of Kiss1r offered protection against steatosis and decreased fibrosis.^102^

#### 1.9.3 KP regulation of the adipose tissue

*Kiss1* mRNA has been found in adipose tissue in rats^99^ and humans.^103^ Fasting has been shown to increase expression of *Kiss1* mRNA in rats, and an HFD decreases it. In women, there is a positive correlation between BMI and *KISS1* mRNA levels in omental (surrounding peritoneal organs) adipose tissue but not in subcutaneous fat.^103^ In vitro studies on mouse 3 T3‐L1 cells and isolated rat adipocytes have revealed expression of *Kiss1* and *Kiss1r* (mRNA and peptide) and explored a possible role of KP on lipid metabolism.^104^ It was found that KP inhibited proliferation, viability, and adipogenesis in 3 T3‐L1 cells and decreased expression of peroxisome proliferator‐activated receptor‐gamma and CCAAT/enhancer‐binding protein beta genes, factors that are involved in the differentiation processes and adipogenesis. Kp‐10 also stimulated lipolysis in 3 T3‐L1 cells and rat adipocytes by increasing expression of perilipin and hormone‐sensitive lipase, as well as modulated glucose uptake and lipogenesis. Additionally, KP decreased glucose uptake and secretion of adiponectin and stimulated secretion of leptin from rat adipocytes.^104^ From these in vitro studies, it was concluded that Kp‐10 may decrease lipogenesis and slightly increase lipolysis. However, there is a need to perform more in vivo experiments on the role of KP on adipose tissue. It was already found that in underfed and fasting male rhesus monkeys there is a stimulatory effect of Kp‐10 on adiponectin but not leptin levels.^105^

As *Kiss1r* is expressed in BAT, KO studies by Tolson et al^106^ explored the role of this expression in relation to obesity. The researchers first showed that global *Kiss1r* KO mice have alterations in body temperature and BAT thermogenic gene expression, factors which could contribute to an obese phenotype. Later using Cre/lox technology they generated conditional *Kiss1r* KO exclusively in BAT (BAT‐*Kiss1r* KO). These mice were not hypogonadal, but both sexes had lower BW and adiposity than controls. However, this phenotype was greater in females. Thus, it was confirmed that the previously observed obesity and decreased metabolism in global *Kiss1r* KOs reflect impaired KP signaling in non‐BAT tissues. This study also added a role for endogenous KP signaling in BAT in modulating metabolism and thermogenesis. However, these experiments were performed on mice and further experiments are needed in different species.

#### 1.9.4 Trihormonal regulatory circuit linking role of KP in regulation of metabolism

Song et al^18^ proposed a trihormonal regulatory circuit between pancreatic α‐cells (secreting glucagon), hepatocytes, and β cells (secreting insulin), identifying a crucial role for KP. According to this hypothesis, liver glucagon receptor activation stimulates insulin secretion by increased hepatic glucose production and hyperglycemia, and liver glucagon action may inhibit insulin secretion by stimulating KP production. Thus, in T2D patients, β cells are exposed to two counteracting stimuli, glucagon‐induced hepatic glucose production, and hyperglycemia stimulation, whereas KP production inhibits glucose‐stimulated insulin secretion. These findings suggest potential for KP antagonism as a therapeutic tool to improve β‐cell function in diabetic patients.^18^ Indeed, it was found that KP‐10 administered to men with T2D and central hypogonadism enabled increased T secretion.^69^

### 1.10 KP alterations both in metabolic and reproductive functions

Jayasena et al observed a massive placenta release of KP into the maternal circulation, suggesting a role for this peptide in pregnancy.^95^ Due to the dramatic rise of circulating levels of KP during healthy pregnancy, it has been proposed as a potential biomarker of placental function. Furthermore, alterations in levels of this peptide are often connected with an increased risk of maternal and fetal complications.^107^ It was also revealed that KP is a placental signal that plays a role in islet adaptation to pregnancy, maintaining maternal glucose homeostasis, acting through the β‐cell Kiss1r. Consequently, it was found that decreased placental KP production and impaired KP‐dependent β‐cell compensation may play an important role in the development of gestational diabetes in humans.^108^

The KP system was also reported to be altered in reproductive organs such as ovaries and testes in animal models of obesity. An HFD administered to female rats at postweaning resulted in a marked suppression of ovarian *Kiss1* mRNA levels. Moreover, in the HFD group, the immunoreactivity of KP was significantly lower in theca cells from antral and preovulatory follicles.^109^ KP and Kiss1r protein expression were reduced in testicular tissues in the diet‐induced obese male mice.^110^

Recently, a role of KP was also expanded on behaviors such as olfactory‐mediated partner preference, sexual motivation, copulatory behavior, bonding, mood, and emotions.^76^ Thus, it would be of interest to study such behaviors in relation to KP in obese and diabetic animals.

In summary, in addition to the central role of KP in the hypothalamus in the regulation reproduction, there are convincing data about the role of this peptide in the regulation of metabolism.

### 1.11 Clinical applications of KP

Reproductive dysfunction and metabolic disturbances are often seen together in conditions such as diabetes, obesity, eating disorders, and PCOS. KP may link reproduction and metabolism by obtaining signals about energy needs at differing stages of development and influencing HPG responses. For example, leptin may regulate metabolic signals on KP as in the ARC, 40% of KP neurons express leptin receptors, whereas GnRH neurons do not.^111^ Although early studies showed direct linkage between KP and leptin, GnRH and leptin connections are more complex and may include a range of other molecules such as ghrelin, NPY, and POMC, as well as involving estradiol, testosterone, progesterone, and stress hormones.^40^ By studying the role of KP in disorders involving reproductive and/or metabolic disturbances, our knowledge of the biology of KP is expanding.

Advances in the field of KP research are represented by a number of clinical trials, which have increased in the last decade. Table 1 shows recent and ongoing KP clinical trials and their status. The main studies are being carried out on kisspeptin 112–121 (or Kp‐10) likely because Kp‐10 is the smallest length needed to stimulate the receptor.^14^ Table 1 summarizes the range of studies that are being conceptualized and the countries that are interested in this field. From this perspective, the focus of the majority of the studies is on fertility issues, or rather conditions associated with infertility (such as PCOS), improving implantation and successful pregnancy. This then correlates to where funding is obtained from to carry out these studies. Furthermore, this table highlights that, to date, there are few published clinical studies on both fertility and metabolism. This suggests that the connectivity between fertility and metabolism is still in its infancy, but it is envisaged that this will change in the future.^119^ As peripherally administered kisspeptin‐54 has been demonstrated to cross the blood–brain barrier^120^ and potentially affect regulation of food intake. Thus, further studies examining the acute and chronic effects of KP on food intake in obese and diabetic patients are needed. It was already found that KP administration to healthy men enhances insulin secretion in response to an intravenous glucose load, without influence on fasting insulin levels or insulin levels in response to delivery of food ad libitum.^119^ This may also open a possibility for a potential therapeutic role for KP treatment in a state of hyperglycemia observed during pregnancy.^121^, ^122^ Finally, it was already proved that KP could be used to enhance both reproductive hormones and insulin levels in obese hypogonadal men with T2D. However, further studies including those that use imaging techniques are needed to explore the role of KP in regulation of metabolism. Such experiments have just begun to emerge. In a study using functional magnetic resonance imaging it was recently found that peripheral administration of KP to healthy men does not alter brain responses to visual food stimuli^123^ or psychometric indices of appetite. However, this study was performed only on men and as was previously discussed, the KP system is sexually dimorphic. Moreover, the odors cue was not taken into account. Finally, in view of their presented findings it would be of interest to study KP effects in obese and T2D patients of different genders.

**TABLE 1:** Recent clinical trials involving KP.

Study and phase	Status	Conditions	Interventions	Results	ClinicalTrials.gov identifier (location)
Metabolism studies					
Evaluation of kisspeptin glucose‐stimulated insulin secretion with oral glucose tolerance Test (Phase I)	R ~ 2024 (*n* = 16)	Metabolic diseases	Kisspeptin	N/A	NCT04958109 (United States)
Evaluation of kisspeptin stimulated insulin with hyperglycemic clamp (Phase I)	R ~ 2024 (*n* = 12)	Metabolic disease	KP‐10	N/A	NCT05456854 (United States)
Evaluation of kisspeptin glucose‐stimulated insulin secretion with physiologic mixed meal tolerance (Phase I)	R ~ 2023 (*n* = 25)	Assessment of beta‐cell responsitivity in healthy women	Kp‐10	N/A	NCT04532801 (United States)
KP‐10 and insulin secretion in men (Phase III)	C 2014 (*n* = 14)	Action on healthy and obese diabetic men	KP‐10	No	NCT03771326 (Pakistan)
Fertility studies					
Detection of kisspeptins and miRNAs in patients with nonviable pregnancy (N/A)	R Prospective (*n* = 433)	Nonviable pregnancy	N/A – diagnostic observational	N/A	NCT03877939 (Spain)
Reproductive hormones during sustained administration of kisspeptin (N/A)	R ~ 2027 (*n* = 76)	Fertility disorders Hypothalamic dysfunction	Kisspeptin	N/A	NCT081924 (United Kingdom)
Opioid Antagonism in Individuals Ascertained Through the Partners Health Care Biobank (Phase I)	R ~ 2025 (*n* = 23)	Reproductive disorders	Kisspeptin 112–121	N/A	NCT04975347 (United States)
Administration of kisspeptin to subjects with reproductive disorders (Phase I)	R ~ 2025 (*n* = 496)	Hypogonadotropic Hypogonadism Kallmann syndrome GnRH deficiency Polycystic Ovarian syndrome Hyperprolactinemia	Kisspeptin 112–121	N/A	NCT00914823 (United States)
Neuropeptides in human reproduction (Phase I)	R ~ 2025 (*n* = 128)	Hypogonadotropic Hypogonadism	Kisspeptin 112–121	N/A	NCT01952782 (United States)
Prolonged pulsatile kisspeptin administration in hypogonadotropic hypogonadism	R ~ 2025 (*n* = 24)	Hypogonadotropic Hypogonadism	Kisspeptin 112–121	N/A	NCT04648969 (United States)
Opioid antagonism in hypergonadotropic hypogonadism (Phase II)	R ~ 2024 (*n* = 23)	Hypogonadotropic Hypogonadism	Kisspeptin	N/A	NCT04975334 (United States)
Kisspeptin administration subcutaneously to patients with reproductive disorders (KASPR) (Phase I)	R ~ 2024 (*n* = 50)	Hypogonadotropic Hypogonadism Hypothalamic Amenorrhea	Kisspeptin 112–121	N/A	NCT05633966 (United States)
Administration of kisspeptin in patients with hyperprolactinemia (Phase II)	R ~ 2023 (*n* = 60)	Hyperprolactinemia Hypogonadism	Kisspeptin 112–121	N/A	NCT02956447 (United States)
Kisspeptin in the evaluation of delayed puberty (Phase I)	R ~ 2023 (*n* = 60)	Delayed Puberty Kallmann Syndrome Hypogonadotropic Hypogonadism GnRH deficiency	Kisspeptin 112–121	N/A	NCT01438034 (United States)
Elucidating kisspeptin physiology by blocking kisspeptin signaling (Phase I)	C 2016 (*n* = 96)	Hypogonadotropic Hypogonadism Healthy postmenopausal women Primary gonadal insufficiency in men	Kisspeptin 112–121	Yes^35^	NCT01438073 (United States)
Age‐dependent changes in the responsiveness of hypothalamic pituitary gonadal axis in men	C 2015 (*n* = 15)	Sterility Reproductive physiological phenomena	Kp‐10	Yes^112^	NCT03315325 (Pakistan)
Link between the sensitivity of kisspeptin signaling and pubertal onset in boys (Phase III)	C 2015 (*n* = 30)	Reproductive physiological phenomena	Kp‐10	Yes^113^	NCT03286517 (Pakistan)
Effects of TAK‐448 in middle‐aged and older men with low testosterone (Phase II)	T 2016 (*n* = 17, intended *n* = 99)	Low testosterone	TAK‐448 (KISS1R agonist, kisspeptin analog)	Partially as terminated early so not analyzed but available in database	NCT02381288 (United States)
A Phase 2a pharmacodynamic study of TAK‐448 in participants with hypogonadotropic hypogonadism (Phase IIa)	T 2015 (*n* = 15, intended *n* = 48)	Hypogonadotropic Hypogonadism	TAK‐448 (KISS1R agonist, kisspeptin analog)	Partially as terminated early. Published in^114^	NCT02369796 (United States)
Serum kisspeptin: A predictive marker for miscarriage or not (N/A)	C 2020 (*n* = 182)	Miscarriage	N/A – observational KP levels	No	NCT03940495 (China)
Tachykinin and Kisspeptin expression in human granulosa and cumulous cells (N/A)	C 2019 (*n* = 236)	Infertility	N/A – observational KISS1 and KISS1R levels	Yes^115^	NCT02877992 (Spain)
Kisspeptin/GPR54 pathway and early puberty (N/A)	C 2019 (*n* = 628)	Precocious puberty	N/A – observational KP levels	Yes^116^	Yes^116^
Kisspeptin levels in early pregnancy (N/A)	C 2019 (*n* = 88)	Early pregnancy Ectopic pregnancy	N/A – diagnostic observational	No	NCT04371991 (Turkey)
Serum kisspeptin levels in infertile women (N/A)	C 2017 (*n* = 90)	Infertility Polycystic ovary syndrome Male factor infertility	N/A – observational	Yes^117^	NCT03018314 (Turkey)
The use of the hormone kisspeptin in in vitro fertilization” treatment	C 2016 (*n* = 175)	Infertility	Kisspeptin, Kisspeptin‐54	Yes^118^	NCT01667406 (United Kingdom)
Metabolism and fertility studies					
Kisspeptin influence on glucose homeostasis (Phase I)	R ~ 2024 (*n* = 413)	Impaired glucose tolerance Hypogonadism	Kisspeptin 112–121	N/A	NCT02953834 (United States)

Abbreviations: C, Completed; GnRH, gonadotropin‐releasing hormone; KP, kisspeptin; N/A, not applicable; R, recruiting; T, terminated.

## 2 CONCLUDING REMARKS

Emerging data from experiments conducted on animals as well as from human clinical trials relating to KP indicate a valuable therapeutic target to treat both metabolic and reproductive dysfunctions. However, it is important to consider differences in gender outcomes related to the KP system. Thus, more research is needed to elucidate the mechanisms of action to develop appropriate therapeutics. There is also recent evidence that KP is involved in emotional behavior including fear, mood, and social and sexual behavior (sexual motivation, copulatory behavior, bonding^76^) that makes sense to enable successful reproduction but may also serve to help control eating disorders and depression.^124^ This is an exciting field, which has yet to reveal its full therapeutic potential.

## FUNDING INFORMATION

Nicola Woods was sponsored through a Biotechnology and Biological Sciences Research Council (BBSRC) Case studentship: grant number BB/F017642/1. Abdullah Alzahrani was funded by the Royal Embassy of Saudi Arabi‐Saudi Cultural Bureau in London. Elpiniki Paspali is sponsored through a University of Strathclyde studentship. Joanna Sliwowska is supported by the National Science Centre in Krakow, Poland (OPUS grant 2015/17/B/NZ4/02021) and the statutory funding (506‐511‐09‐00) from the Faculty of Veterinary Medicine and Animal Science, Poznan University of Life Sciences, Poland to Joanna Sliwowska.

## DISCLOSURE

No disclosures.
